# Supplementary material for: Patients’ and healthcare professionals’ perspectives towards technology-assisted diabetes self-management education. A qualitative systematic review
Source: PLoS One. 2020 Aug 17;15(8):e0237647. doi: 10.1371/journal.pone.0237647 (PMC7430746; doi:10.1371/journal.pone.0237647)
Supplement: S1 Appendix — (DOCX) [file pone.0237647.s002.docx]

**S1 Appendix: Medline Search**

1 exp diabetes mellitus, non-insulin-dependent/

2 exp insulin resistance/

3 impaired glucose toleranc$.tw.

4 glucose intoleranc$.tw.

5 insulin$ resistanc$.tw.

6 exp obesity in diabetes/

7 (obes$ adj diabet$).tw.

8 (MODY or NIDDM).tw.

9 (non insulin$ depend$ or noninsulin$ depend$ or noninsulin?depend$ or non insulin?depend$).tw.

10 ((typ$ 2 or typ$ II) adj diabet$).tw.

11 ((keto?resist$ or non?keto$) adj diabet$).tw.

12 ((adult$ or matur$ or late or slow or stabl$) adj diabet$).tw

13 (insulin$ defic$ adj relativ$).tw. or pluri?metabolic$ syndrom$.tw.

14 or/1-13

15 Exp Health Education/

16 exp Professional-Patient Relations/

17 educat*.tw.

18 Program*.tw.

19 promotion*.tw.

20 counsel*.tw.

21 information.tw.

22 or/15-21

23 exp Self Care/

24 self-manag$.tw.

25 self manag$.tw.

26 manag*.tw.

27 self-car$.tw.

28 self car$.tw.

29 selfcar$.tw.

30 self-administ$.tw.

31 self administ$.tw.

32 (patient$ adj3 (focus$ or participat$ or centr$ or center$ or empower$ or support$ or collaborat$ or co-operat$ or cooperat$ or educat$ or service$ or recipient)).tw.

33 or/23-32

34 interviews as topic/ or focus groups/ or narration/ or qualitative research/

35 ((("semi-structured" or semistructured or unstructured or informal or "in-depth" or indepth or "face-to-face" or structured or guide) adj3 (interview$ or discussion$ or questionnaire$)) or (focus group$ or qualitative or ethnograph$ or fieldwork or "field work" or "key informant")).ti,ab.

36 34 or 35

37 14 AND 22 AND 33 AND 36
