# Supplementary material for: Patients’ and healthcare professionals’ perspectives towards technology-assisted diabetes self-management education. A qualitative systematic review
Source: PLoS One. 2020 Aug 17;15(8):e0237647. doi: 10.1371/journal.pone.0237647 (PMC7430746; doi:10.1371/journal.pone.0237647)
Supplement: S2 Appendix — (DOCX) [file pone.0237647.s003.docx]

**Appendix 2: CASP qualitative checklist**

| **Paper** | **1. Was there a clear statement of the aims of the research?** | **2. Is a qualitative methodology appropriate?** | **3. Was the research design appropriate to address the aims of the research?** | **4. Was the recruitment strategy appropriate to the aims of the research?** | **5. Was the data collected in a way that addressed the research issue?** | **6. Has the relationship between researcher and participants been adequately considered?** | **7. Have ethical issues been taken into consideration?** | **8. Was the data analysis sufficiently rigorous?** | **9. Is there a clear statement of findings?** | **Overall assessment*** |
| --- | --- | --- | --- | --- | --- | --- | --- | --- | --- | --- |
| S. E. Mitchell et al., 2014 | Yes | Yes | Yes | Yes | Yes | Cannot tell | Yes | Yes | Yes | No/very minor concerns |
| C. H. Yu et al., 2014 | Yes | Yes | Yes | Yes | Yes | Cannot tell | Yes | Yes | Yes | No/very minor concerns |
| C. H. Yu et al., 2014 | Yes | Yes | Yes | Yes | Yes | Yes | Yes | Yes | Cannot tell | No/very minor concerns |
| N. Patel et al., 2015 | Yes | Yes | Yes | Yes | Yes | Cannot tell | Yes | Cannot tell | Cannot tell | Moderate considerations |
| M. Hofmann et al., 2016 | Yes | Yes | Yes | Cannot tell | Yes | Cannot tell | Yes | Yes | Yes | Minor concerns |
| J. Jafari et al., 2016 | Yes | Yes | Yes | Cannot tell | Yes | Cannot tell | Yes | Yes | Yes | Minor concerns |
| S. M. Andrews et al., 2017 | Yes | Yes | Yes | Yes | Yes | Cannot tell | Yes | Yes | Yes | No/very minor concerns |
| D. D. Maglalang et al., 2017 | Yes | Yes | Yes | Yes | Yes | Yes | Cannot tell | Yes | Yes | No/very minor concerns |
| J. Hall et al., 2018 | Yes | Yes | Yes | Cannot tell | Yes | Cannot tell | Yes | Cannot tell | Yes | Moderate considerations |
| L. Poppe et al., 2018 | Yes | Yes | Yes | Cannot tell | Yes | Cannot tell | Yes | Cannot tell | Yes | Moderate considerations |
| K. M. Smith et al., 2018 | Yes | Yes | Yes | Cannot tell | Yes | Cannot tell | Yes | Yes | Yes | Minor concerns |
| D. K. King et al., 2012 | Yes | Yes | Yes | Cannot tell | Yes | Cannot tell | Cannot tell | Yes | Yes | Moderate considerations |
| Pal K et al., 2018 | Yes | Yes | Yes | Yes | Yes | Cannot tell | Yes | Yes | Yes | No/very minor concerns |
| L Desveaux et al., 2018 | Yes | Yes | Yes | Yes | Yes | Yes | Yes | Yes | Yes | No/very minor concerns |
| L Kelly et al., 2018 | Yes | Yes | Yes | Yes | Yes | Cannot tell | Yes | Yes | Yes | No/very minor concerns |

*The overall assessment was rated on a scale: ‘no/very minor concerns’, ‘minor concerns’, ‘moderate considerations’, and ‘serious concerns’
