# Supplementary material for: Patients’ and healthcare professionals’ perspectives towards technology-assisted diabetes self-management education. A qualitative systematic review
Source: PLoS One. 2020 Aug 17;15(8):e0237647. doi: 10.1371/journal.pone.0237647 (PMC7430746; doi:10.1371/journal.pone.0237647)
Supplement: S3 Appendix — (DOCX) [file pone.0237647.s004.docx]

| **Line-by-line Coding** | **Descriptive Themes** | **Analytical Themes** |
| --- | --- | --- |
| “SMS is an effective way, and three times per week is good. Once a week may be forgotten, and every day could be boring. One should consider the psychological aspects of its effect." Patient 6, male, 58 years (1) | Effective reminders for use | Easy accessibility to the interventions as a facilitator for use |
| “Constantly reminding me about the things that we need to be aware of. Most of us know but the Online Companion was a good reminder and got me thinking of things that I need to constantly do (some that we conveniently forget).” [3B55, 58-year old woman] (2) |  |  |
| “Sometimes I do not think about the fact that I am diabetic but then you receive an e-mail that you need to fill out the website. It awakes the subconscious idea that you need to move more. I feel like they are reminders that keep you awake.” (Male, 61 years old) (3) |  |  |
| “The website itself is easy to navigate. And I think that list is very good and I’ve found that every time I have looked, [the answer] could typically could fall into one of those categories. The answer might not be there, but I know where to start to look.” [2B09, 47-year old woman] (2) | Easy usability of the interventions |  |
| “Yes, it is easy to use and that is nice. You only need to read one thing, not a whole text that you need to go through. These are short things, short questions and it goes well.” (Male, 73) (3) |  |  |
| “It was quite easy for somebody who doesn’t know about anything, so it was quite easy and a very, very simple way to explain the benefits of the insulin.” (Patient) (4) |  |  |

**S3 Appendix: Examples of theming syntheses according to Thomas and Harden**

1. Jafari J, Karimi Moonaghi H, Zary N, Masiello I. Exploring educational needs and design aspects of internet-enabled patient education for persons with diabetes: a qualitative interview study. BMJ Open. 2016;6(10):e013282.

2. Yu CH, Parsons JA, Mamdani M, Lebovic G, Hall S, Newton D, et al. A web-based intervention to support self-management of patients with type 2 diabetes mellitus: effect on self-efficacy, self-care and diabetes distress. BMC Medical Informatics and Decision Making. 2014.

3. Poppe L, Crombez G, Bourdeaudhuij I, Mispel C, Shadid S, Verloigne M. Experiences and Opinions of Adults with Type 2 Diabetes Regarding a Self-Regulation-Based eHealth Intervention Targeting Physical Activity and Sedentary Behaviour. International Journal of Environmental Research and Public Health. 2018;15.

4. Patel N, Stone M, Hadjiconstantinou M, Hiles S, Troughton J, Martin-Stacey L, et al. Using an interactive DVD about type 2 diabetes and insulin therapy in a UK South Asian community and in patient education and healthcare provider training. Patient Education and Counseling. 2015;98:1123-30.

**References**
