# Supplementary material for: Patients’ and healthcare professionals’ perspectives towards technology-assisted diabetes self-management education. A qualitative systematic review
Source: PLoS One. 2020 Aug 17;15(8):e0237647. doi: 10.1371/journal.pone.0237647 (PMC7430746; doi:10.1371/journal.pone.0237647)
Supplement: Attachment — Submitted filename: Reviewer feedback_PONE-D-20-02347_26Mar20.pdf [file pone.0237647.s005.pdf]

Reviewer comments

Reviewer: Willem Odendaal

Manuscript ID: PONE-D-20-02347
Title: Perspectives Towards Technology-assisted Diabetes Self-Management Education. A Qualitative Systematic Review

Date: 26/03/20

Dear Editor and Authors

In my view, the manuscript covers an important topic, worthy to publish. However, I recommend that the authors address the major revisions, before accepting it for publication. There are also in-text revisions, which if addressed, I believe will improve the manuscript.

Major revisions

1. Provide a rationale for why the different technologies are lumped together. Is there evidence that technology type is not a variable to consider in understanding user perceptions and experiences?
   - Is an App on a phone (ref 22) similar enough to a website (in nine of the 13 included studies), and a website similar enough to telehealth (ref 23) to assume that blanket conclusions can be drawn across the types of technology?
   - Linked to this is a blanket statement (line 180) that "Some had issues with the cost of the intervention". If it applied to all the technologies, say so, or tell the reader which technologies raised these concerns.
2. It appears that findings from Biernatzki et al (Information needs in people with diabetes mellitus: a systematic review. *Syst Rev* **7,** 27: https://doi.org/10.1186/s13643-018-0690-0) suggest that there may be differences between diverse diabetes populations regarding information needs which in turn may inform patients' technology preferences. Please provide a rationale for not using diverse patient groups as a variable in perceptions and experiences.
3. Points 1 and 2 should inform a more nuanced Discussion and Conclusions.
4. Please link this review with effectiveness reviews. This should be addressed in the Background and Discussion, with a deliberate reference on how they complement each other. It will be interesting to know if their qualitative results can help understand effectiveness outcomes. Are the quantitative results of the effectiveness reviews, Reference 32 and 39, not of any relevance to understand the importance of patients' perceptions and experiences?
5. Provide a rationale why they excluded the perceptions and experiences of informal caregivers such as family members or lay health workers. I'm not expecting them to be included, but my view is that they play as big a part in the self-management of a chronic condition, as healthcare workers, and hence their voices are important?
6. Provide more information regarding their methodology:
   - Provide the screening tool, i.e. the inclusion / exclusion criteria, in particular the participants and technology.
   - Did you have criteria regarding publication date and setting?
   - Detail who did the screening: was it the same two authors for title/abstract and full texts respectively, and if Yes, why?

- Add more detail about the coding process: (i) who did it, and (ii) did the referred two authors coded each paper independently and then compared, or did they sat together, or did they do one or two together to draft a coding list, or did they use a different method.
- Substantiate their claim that theirs is the 1st qualitative systematic review by providing evidence that they have searched systematic review data bases, such as:
  - Cochrane library: https://www.cochranelibrary.com/
  - Epistemonikos: https://www.epistemonikos.org/
  - McMasters Health evidence: https://www.healthevidence.org/

7. Please add quotes strategically that will clarify some of the results.
8. Evidence that the manuscript was proof-read for language. There are too many instances, such as the following, that need a revision to clarify its meaning.
   Lines 33-34: "Stakeholders had mixed views towards features of the technology-assisted interventions and patients' personal qualities and providers' concerns that affected their use of the interventions."
   Lines 309-10: "Since patients were unable to fulfill their preferred method of social support online and felt that these interventions were ineffective in creating peer support."

Important in-text revisions

1. *Title:* Add wording to the title that will make it clear that the review is about patients' and healthcare workers' perspectives

2. At times you refer to healthcare workers (HCWs) as "professionals" (line 39); "various healthcare providers", (line 61) which could include all cadres; "Nurses, nurse managers and patient care technicians" (line 247); and "hospital workers" (line 252).
   - Decide on a terminology, describe it clearly and use it consistently.

3. The Discussion in lines 42-3 looks like a conclusion, and there is no conclusion in the abstract.

4. Add a space between the last word and in-text citation of a reference.

Line 21: Add whose perspectives you are referring to.

Line 23: Add HCWs to the sentence, else it seems it is only about patients.

Line 33: Clarify who the stakeholders are, keeping in mind that they may include people other than patients and HCWs

Line 38: At first, I thought of "Community support" as in the communities where patients live, but in the manuscript it is about fellow patients. Clarify this in line 38.

Line 55: Add a reference.

Line 84: Clarify "caregivers": is it only HCWs or does it include family members too?

Line 86: Add a reference.

Line 88: Clarify "disruptive".

Line 96: Clarify “stakeholders”.

Line 111: What software did you use to screen, and how did you resolve the differences?

Line 118: Did you include original, non-peer reviewed papers?

Line 119: What is the difference between a ‘dissertation’ and ‘thesis’?

Line 120: Add the software you used.

Line 121: “a senior author” could be a content expert outside the author team?

Line 132: Which authors?

Line 138: Who was the “independent 3rd author?” ‘Independent’ from the appraisal but part of the team?

Table 1:

- Clarify how ‘web-based’ and ‘web-site' differs in refer 18.
- Provide a rationale why you choose age as a variable and not something like SES or education? Is there literature that links attitude towards technology with age?

Lines 174-76: Add references and “with better health literacy will be able to …” should rather be in the Discussion or rephrased as a result.

Lines 181-82: Is this not a repeat of line 179-80?

Line 185: It can be understood as information about the intervention, for example how to use it. My read is that you rather mean the “information made available through the intervention.” If that is the case, rephrase the sentence.

Lines 196-97: See my query regarding line 185.

Line 208: Add reference.

Lines 209-10: How is it different to lines 190-91?

Line 213: What value is it adding as it repeats lines 216-17?

Line 215: “They felt …”: Assuming these are the older patients, do you have data on what the younger patients themselves said?

Line 227: See my query line 185.

Line 244: Add reference.

Line 248: This theme is about health workers and not patients.

Line 249: Who are “they”: patients and nurses?

Line 249: Provide an example of “integration with the workflow”.

Line 253: Were the technology used in the hospital?

Line 260: A repeat of lines 188-89?

Lines 261-62: Reads like a conclusion and not result.

Lines 303-05: Is this the authors’ opinion or patients’? If the former, move it to the Discussion.

Lines 356-62: This is just a repeat of results, and should rather be discussed in relation to the literature on these issues.

Line 369: Please provide practical examples of how this support can be provided.

Line 360: Clarify “providers”; see Query 1 above.

Lines 373-78: Please link this with existing literature.

Limitations:

- Qualitative research is not about numbers, but all the included studies were small. I suggest it as a limitation in a more general sense of studies exploring the use of technology.
- How many of the included studies reported on technology that have become standard care? My sense from the titles are that many of the technologies were being piloted. If that is indeed the case, I think it to be a limitation, because piloting is generally a world apart from standard practice, with the former often being better resourced with project staff who want to make the technology work.
- Nine of the 13 included studies are about web-based technology or website technology. Is this not skewing the results?
- Clarify who the “administrators” are?

Line 398: Is there evidence in the results that HCWs were involved in delivering the DSME technology?

Lines 400-03: Is it not that the “positive engagement” resulted from all the issues listed, and not preceded it?

Line 409: This does not make sense as there are no abbreviations provided

S1 Appendix: I’m missing “technology” in the search terms. If it is missing, is there a reason for not including it?

In-text suggestions

Line 36: Use a synonym for “impetus”.

Line 45: “was” rather than “is”, that is assuming that the study came to an end.

Lines 69-72: Too long sentence.

Lines 126-131: Add an example of the coding > descriptive themes > analytic themes as an appendix.

Line 143: Add the number of studies to the countries.

Table 1

- Include the reference number below the author name
- What about adding an appendix with detail of how the technology worked? think it will help the reader to better understand the results
- Be consistent in 'interviews' in the Methodology column. Sometimes you used 'interview'.
- 1st time use of "HCP" requires writing in full.

Line 184: Rather just "Mixed views on the technology ~~provided intervention~~"

Line 201: "for diabetes self-management" seems redundant to me.

Lines 213-17: Does it not belong to "*Accessibility of the Intervention*"?

Line 230: Was there another application as to real life? Consider some like: 'Applying self-management interventions'.

Line 254: Consider "perceptions" rather than "emotions".

Line 274: "had" rather than "has"?

Line 318: Will "perceptions" not work better than "emotions"?

Lines 322, 362: "previous literature" is not scholarly.

Line 365: "hiccups" not scholarly.

Lines 381-82: Consider adding "… using standardised quantitative measures in larger scale studies, to better …".

Lines 382-86: You are saying that mixed-methods are required, but 5/13 (38%) included studies used mixed methods. Why is this not enough?

Line 385: How is "biochemical" and "physiological" different?

Line 405: Which "group" are you referring to? It reads as if it is not diabetic patients but a group of these patients.

Fig 1: 36 studies were excluded because of quantitative analysis. What about the data collection? Consider using "quantitative methods".

Appendix 2: Consider adding a column that details your overall assessment: 'no/very minor concerns' or 'minor concerns' or 'moderate concerns' or 'serious concerns' about the methodological rigour of a study.
