# Supplementary material for: Patients’ and healthcare professionals’ perspectives towards technology-assisted diabetes self-management education. A qualitative systematic review
Source: PLoS One. 2020 Aug 17;15(8):e0237647. doi: 10.1371/journal.pone.0237647 (PMC7430746; doi:10.1371/journal.pone.0237647)
Supplement: Attachment — Submitted filename: Response to Reviewers_14-07-2020.docx [file pone.0237647.s007.docx]

**Response to Reviewers**

**Title:** Patients’ and Healthcare Professionals’ Perspectives Towards Technology-assisted Diabetes Self-Management Education. A Qualitative Systematic Review

**Manuscript ID**: PONE-D-20-02347R1

**Comments from Reviewer #3**

| **Reviewer’s Comments** | **Response to Reviewers** | **Lines** |
| --- | --- | --- |
| Thanks for your comprehensive response to my comment.  A small matter:  Please explain to readers not familiar with technology jargon, what “disruptive technologies” mean. My understanding is that it refers to innovate technologies that create a "new market and value network and eventually disrupts an existing market and value network, displacing established market-leading firms, products, and alliances". If this is indeed what you meant with 'disruptive' please add, or if you have a different definition, please add that. | Dear Reviewer, thank you for the comment. We have added to the manuscript, detailing the meaning of disruptive technology in the context of diabetes self-management education. | Lines 89-93 |
